# Long-term Relationships between Cholinergic Tone, Synchronous Bursting and Synaptic Remodeling

**DOI:** 10.1371/journal.pone.0040980

**Published:** 2012-07-23

**Authors:** Maya Kaufman, Michael A. Corner, Noam E. Ziv

**Affiliations:** 1 Department of Physiology and Biophysics and Rappaport Institute, Technion Faculty of Medicine, and Network Biology Research Laboratories, Lorry Lokey Center for Life Sciences and Engineering, Haifa, Israel; 2 The Netherlands Institute for Brain Research, Amsterdam, The Netherlands; University of Nebraska Medical Center, United States of America

## Abstract

Cholinergic neuromodulation plays key roles in the regulation of neuronal excitability, network activity, arousal, and behavior. On longer time scales, cholinergic systems play essential roles in cortical development, maturation, and plasticity. Presumably, these processes are associated with substantial synaptic remodeling, yet to date, long-term relationships between cholinergic tone and synaptic remodeling remain largely unknown. Here we used automated microscopy combined with multielectrode array recordings to study long-term relationships between cholinergic tone, excitatory synapse remodeling, and network activity characteristics in networks of cortical neurons grown on multielectrode array substrates. Experimental elevations of cholinergic tone led to the abrupt suppression of episodic synchronous bursting activity (but not of general activity), followed by a gradual growth of excitatory synapses over hours. Subsequent blockage of cholinergic receptors led to an immediate restoration of synchronous bursting and the gradual reversal of synaptic growth. Neither synaptic growth nor downsizing was governed by multiplicative scaling rules. Instead, these occurred in a subset of synapses, irrespective of initial synaptic size. Synaptic growth seemed to depend on intrinsic network activity, but not on the degree to which bursting was suppressed. Intriguingly, sustained elevations of cholinergic tone were associated with a gradual recovery of synchronous bursting but not with a reversal of synaptic growth. These findings show that cholinergic tone can strongly affect synaptic remodeling and synchronous bursting activity, but do not support a strict coupling between the two. Finally, the reemergence of synchronous bursting in the presence of elevated cholinergic tone indicates that the capacity of cholinergic neuromodulation to indefinitely suppress synchronous bursting might be inherently limited.

## Introduction

Cholinergic neuromodulation plays key roles in processes observable at levels ranging from cellular biophysics to behavior, and on time scales ranging from sub-seconds to circadian rhythms. For example, tightly timed cholinergic neuromodulation plays important roles in the regulation of multiple forms of synaptic plasticity (e.g. [1]–[5]). At the other end of the spectrum, changes in cholinergic tone occurring over circadian time scales play crucial roles in regulating major arousal states as well as cortical activity patterns that characterize these states ([6], [7], reviewed in [8], [9]). Specifically, cholinergic tone is relatively high during waking, particularly high during rapid eye movement (REM) sleep periods, and relatively low during non-REM sleep [8], [9].

During late prenatal and early postnatal development, REM sleep is a predominant brain state, and thus, presumably much of brain circuitry development and refinement occurs in a regime of elevated cholinergic tone. Indeed, REM sleep has been shown to play instrumental roles in the maturation of brain circuitry and connectivity (reviewed in [10]) and in synaptic consolidation later in life (reviewed in [11]). Moreover, lesions of cholinergic afferents (mainly from the basal forebrain) have been shown to delay cortical neuronal development, alter cortical cytoarchitecture [12], and suppress cortical map plasticity associated with various sensory deprivation paradigms [13], in particular, forms of cortical plasticity driven by behavioral experience [14]. Presumably, these large scale changes in cortical connectivity are associated with substantial synaptic remodeling, yet to date, relationships between slow changes in cholinergic tone and synaptic remodeling remain largely unknown.

Recent, as well as older studies [15] indicate that wake-sleep cycles are also associated with global changes in synaptic properties. Specifically, electrophysiological, morphological, and biochemical studies in rodents [16]–[21], fish [22] and flies [23]–[25] suggest that waking and sleep are associated with increases and reductions, respectively, in synapse number, synaptic size as well as biochemical and electrophysiological measures of synaptic strength. Given the many physiological differences between waking and sleep brain states, the underlying basis of such changes in synaptic properties is likely to be compound and multifaceted. However, changes in neuromodulator levels during waking and sleep, including those of acetylcholine, are likely to play important roles.

To evaluate how changes in cholinergic tone affect synaptic remodeling over circadian time scales, an experimental system is needed that allows for experimental manipulation of cholinergic tone while continuously recording the structural dynamics of individual synapses over long time scales. Moreover, as cholinergic tone strongly affects network activity characteristics both *in vivo* [6]–[9] and *in vitro* [26]–[29], and given the ability of bioelectric activity *per se* to affect synaptic remodeling, simultaneous monitoring of network activity is required to discern the direct contributions of cholinergic modulation from effects mediated by changes in network activity levels or patterns.

Here we describe the use of such a system [30] to address the following questions: How is synaptic remodeling affected over long time scales by experimental manipulations of cholinergic tone? How are network activity characteristics affected by these manipulations in the same preparations? How do these manipulations affect the rules that govern synaptic remodeling? How are cholinergic-induced changes in synaptic remodeling related to cholinergic-induced changes in network activity properties?

## Results

### Carbachol Application Drives Synaptic Growth

**Figure 1:**
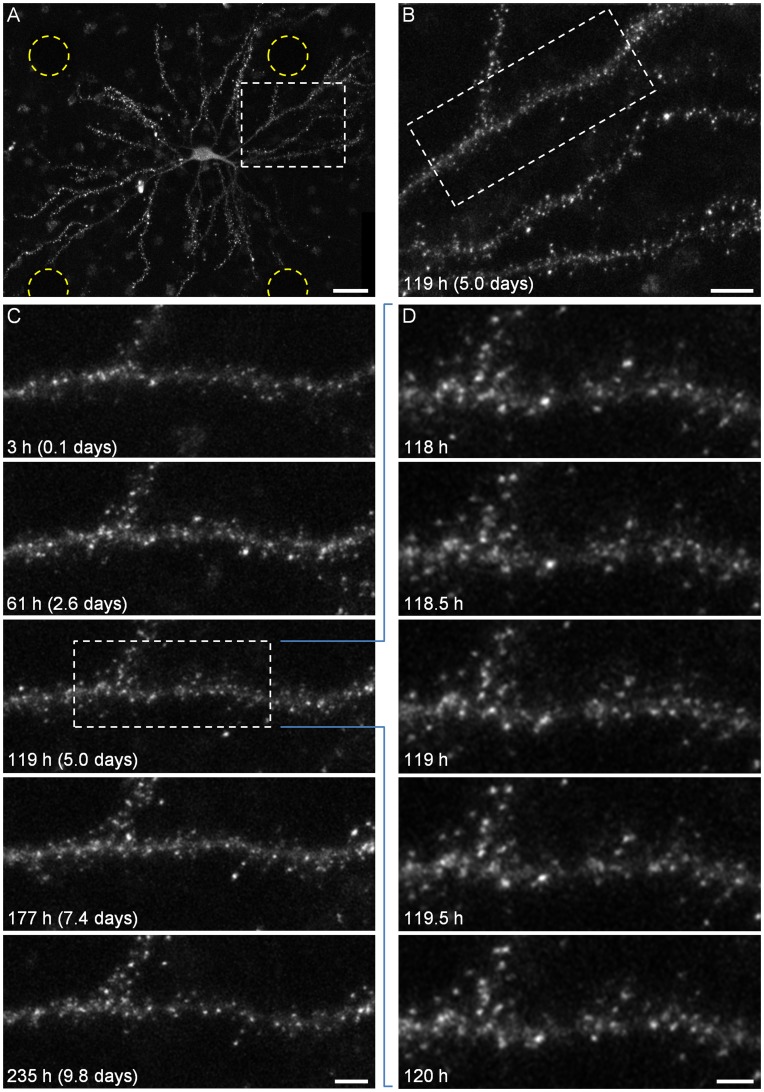
Long term imaging of postsynaptic sites. ***A***) A single neuron expressing PSD-95:EGFP. Fluorescent puncta represent postsynaptic sites formed on dendritic spines and shafts. The positions of four embedded electrodes in the field of view are marked with yellow circles. ***B***) An enlarged portion of the image in *A* (region enclosed in rectangle) showing the typical field of view (site) imaged in these time-lapse recordings. ***C***) A 10-day time-lapse series (30 min intervals, or 48 images/day) of the dendrite enclosed in a rectangle in *B*. Only a small subset of the data is shown here. ***D***) Magnification of region enclosed in a rectangle in *C*, demonstrating the actual temporal and spatial resolution of imaging data collected in these experiments, enabling the tracking of many individual postsynaptic puncta. All images in panels *B-D* are maximal intensity projections of 10 images collected at 10 focal planes spaced 0.8 µm apart. Bars *A*, 30 µm; *B*, 10 µm; *C*, 5 µm, *D*, 3 µm.

In the present study we examined long-term relationships (many hours and days) between cholinergic tone and the remodeling of glutamatergic synapses, the most common type of excitatory synapse in the mammalian CNS. Glutamatergic synapses are typically formed on dendritic spines, minute protrusions that extend from the dendritic shaft, the remodeling of which is widely believed to reflect changes in synaptic strength [31]. To visualize (postsynaptic) remodeling of excitatory synapses, we expressed an enhanced green fluorescent protein (EGFP)-tagged variant of postsynaptic density protein 95 (PSD-95:EGFP). PSD-95 is the major postsynaptic scaffold protein of glutamatergic synapses and is thought to cluster NMDA receptors at postsynaptic sites. Furthermore, through interactions with transmembrane AMPA receptor regulatory proteins, PSD-95 controls the number of AMPA receptors within the postsynaptic membrane (reviewed in [32]). Therefore, to a first approximation, PSD-95:EGFP fluorescence can serve as a proxy of synaptic strength [31]. More conservatively, changes in PSD-95:EGFP fluorescence reflect synaptic remodeling and changes in spine head size (which will be referred to hereafter as synaptic size). PSD-95:EGFP was expressed in a small number of neurons in each dish by means of a third-generation lentiviral expression system as we previously described [30]. As shown in Figure 1, PSD-95:EGFP assumed a punctate appearance, with puncta commonly located at the tips of dendritic spines. As previously demonstrated [30], [33], [34], the large majority of such puncta represent *bona fide* synapses.

To follow how experimental manipulations of cholinergic tone affect excitatory synaptic remodeling, we performed the following experiments: cortical neurons grown in MEA dishes (maintained in culture for at least 18 days) were mounted on a custom built confocal laser scanning (inverted) microscope, and provided with optimal environmental conditions required for long-term recordings (see Materials and Methods). Images (9 to 11 Z-sections) of neurons expressing PSD-95:EGFP were collected automatically from 3–12 fields of view (or sites; ∼95×70 µm in size) at 30 min intervals with each site representing a portion of a neuron’s dendritic arbor (Figure 1B). After 2–4 days of baseline imaging, Carbachol (CCh), a non-hydrolysable analog of acetylcholine, was added directly into the dish and to the perfusion media reservoir (to ensure both abrupt and continuous exposure to the agonist) and recordings were continued for at least two additional days (often longer). Network activity was recorded simultaneously from the MEA throughout all experiments as detailed later. Typically, combined imaging/electrophysiological experiments were carried out for one week or more, with preparations showing no signs of deterioration or cell death, over these and even longer periods (Figure 1, Video S1; see also [30]).

**Figure 2:**
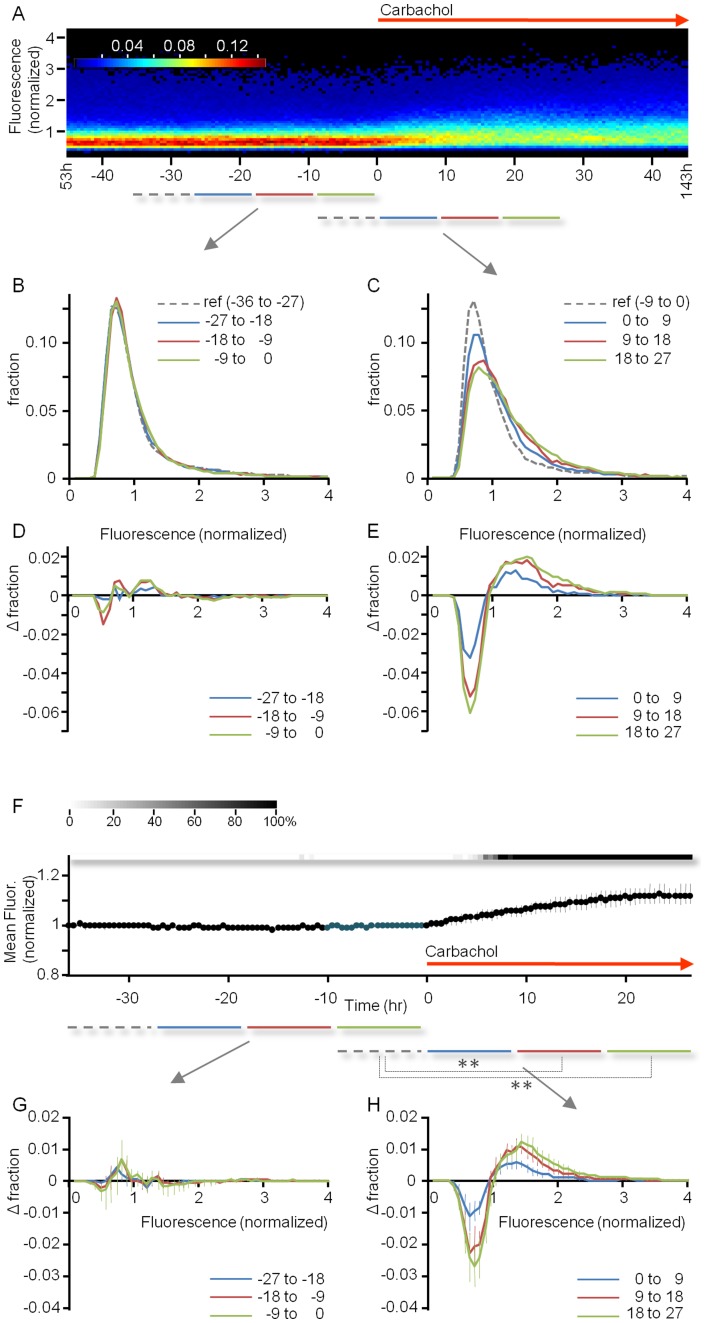
CCh causes a gradual increase in mean synaptic size and a broadening of synaptic size distributions. ***A***) Example of one experiment. CCh (20 µM) was added to the dish and perfusion media 98 hours after the beginning of the experiment. Panel shows color-coded distributions of fluorescence values of PSD-95:EGFP puncta, for each 0.5 hour time-lapse interval, normalized to the average puncta fluorescence over the 45 hour time window before CCh application. Histograms represent pooled data from all 5 sites in one experiment (same experiment as in Figure 3). ***B,C***) Distributions from the experiment shown in *A*, averaged over 9 hour windows (shown as horizontal lines below *A*), 4 windows before and 3 windows after CCh application. ***D,E***) Subtraction of a reference distribution (dashed lines in *A–C*) from each of the subsequent distributions. ***F***) Mean fluorescence of PSD-95:EGFP puncta from 6 experiments (36 neurons, 42 sites). Data is shown from 36 hours before to 27 hours after CCh application. Data was normalized separately in each experiment to the average puncta fluorescence of the 36 hour time window before CCh application. To examine the statistical significance of these changes, a paired two tailed t-test was performed for fluorescence values of each time point and each of the 20 pre-CCh time points (dark blue). The grayscale bar above the graph indicates what percentage (legend at the left top) of such tests resulted in statistically significant differences (p<0.05). ***G,H***) Subtraction of reference distributions as in *D* and *E* for the pooled data of these 6 experiments. Asterisks indicate that >99% of Kolmogorov–Smirnov tests performed for each time point in a particular 9 hour time window were significantly different (p<0.01) from each distribution in appropriate reference windows. Data in *F-H*, mean ± SEM.

We first addressed how CCh affects the distribution of synaptic sizes at the population level, or, more precisely, the distribution of fluorescence values of individual PSD-95:EGFP puncta. To that end, fluorescence values of individual PSD-95:EGFP puncta were collected programmatically at consecutive time points, and distributions and means of these values were computed (Figure 2). As we previously showed ([30; see also [35]) these distributions are highly skewed, and remain quite stable for days (Figure 2A). The application of CCh, however, caused a gradual rightward broadening of these distributions over the next ∼24 hours, after which they stabilized and settled at new values (Figure 2A). CCh-related broadening of PSD-95:EGFP puncta fluorescence distributions become even more apparent when comparing distributions averaged across 9 hour time windows (Figures 2B,C) or when these were compared after the subtraction of reference distributions (Figures 2D,E). As mentioned above, CCh was present continuously from the moment of application onward, as it was added to both the dish and the perfusion media reservoir.

Data pooled from 6 experiments (36 neurons, 42 sites) shows that CCh application was followed by a gradual increase in mean PSD-95:EGFP puncta fluorescence (Figure 2F) and by a shift of PSD-95:EGFP puncta fluorescence distributions toward higher values (Figures 2G,H). In contrast, CCh had no consistent effect on synaptic counts in the same experiments (data not shown). These experiments thus indicate that chronic elevations of cholinergic tone can drive a gradual increase in excitatory synaptic sizes.

### Suppression of Synchronous Bursting by Carbachol

**Figure 3:**
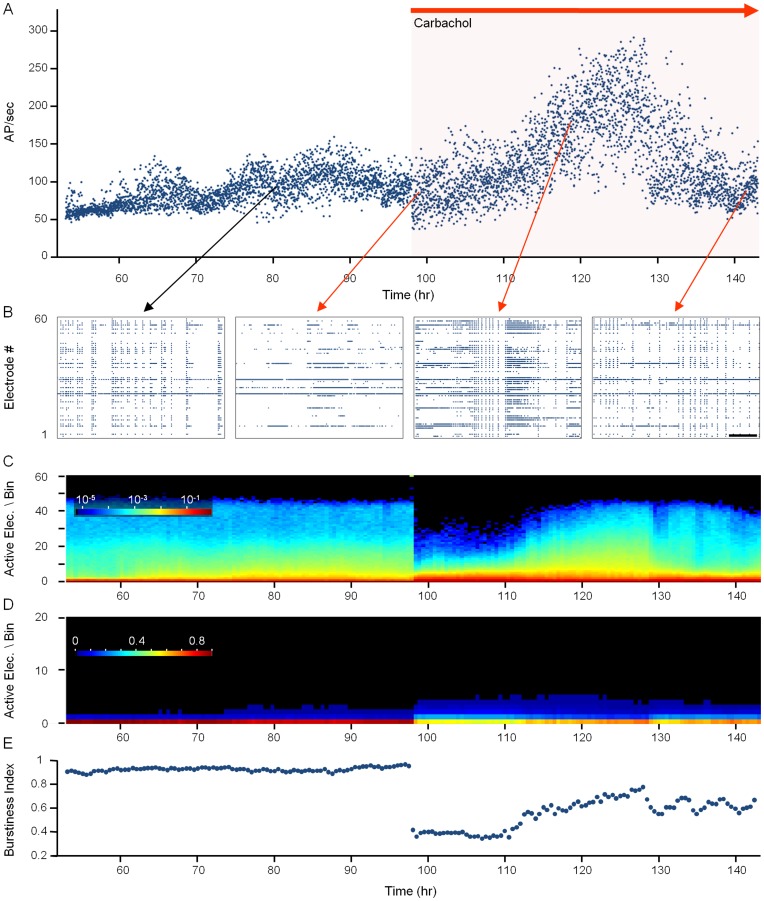
CCh suppresses synchronous bursting in *ex-vivo* networks of cortical neurons. Example of one experiment (same experiment as that shown in Figure 2A–E). ***A***) Spontaneous activity recorded from a network of cortical neurons growing on an MEA dish. A 90 hour time window is shown here, starting 53 hours after mounting of the preparation on the combined MEA recording/imaging system. Activity is expressed as action potentials (measured from all 59 electrodes) per second, averaged over 1 min bins. 98 hours after the beginning of the experiment CCh, (20 µM) was added to the dish and the perfusion media. ***B***) Examples of one-minute long raster plots of activity recorded from 59 electrodes at 80, 99, 118, 142 hours. Note the near elimination of synchronous bursts immediately after CCh application (99 h) Scale bar  = 10 sec. ***C,D***) Histograms showing the distribution of *<number of active electrodes*>/*<10* *msec bin*>. CCh reduced the fractions of bins characteristic of synchronous bursting activity, that is, bins in which most electrodes were active and bins in which no electrodes were active. The histograms are shown in logarithmic (*C*) and linear scales (*D*). ***E***) Changes in the Burstiness Index [39] caused by CCh.

**Figure 4:**
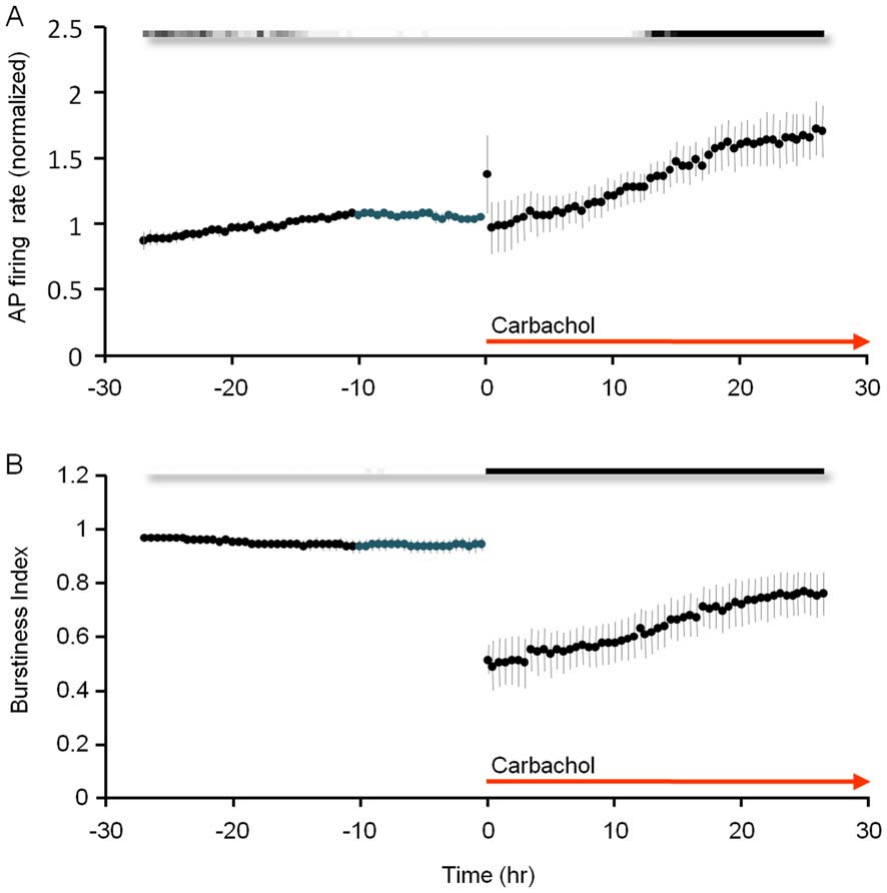
CCh suppresses synchronous bursting while preserving overall activity levels. Pooled data from 6 experiments, 27 hours before and 27 hours after CCh application. ***A***) Firing rates recorded from all 59 electrodes, normalized to average firing rates during the 27 hour time window before CCh application). ***B***) Burstiness index. Grayscale bars indicate statistical significance as in Figure 2F. All data points - mean ± SEM.

As mentioned above, activity was recorded in all experiments through the 59 electrodes of the MEA dish, allowing us to determine how CCh applications affected network activity. Before CCh application, and in agreement with many prior reports (e.g. [36]–[41] reviewed in [42]), the networks of cultured cortical neurons exhibited complex patterns of spontaneous activity, composed mainly of network-wide periods of synchronous bursting occurring once every 1 to 10 seconds, separated by periods of nearly complete quiescence or sparse, asynchronous action potentials. Following CCh application, these activity patterns were strongly altered. This is illustrated by the experiment shown in Figure 3 (same experiment as Figure 2A–E). CCh (20 µM), applied 98 hours (∼4 days) after the beginning of the experiment abruptly reduced the synchronous nature of network activity, as evident in raster plots of raw data (Figure 3B, second panel from left) and in measures of network synchronicity described below. In contrast, overall activity levels, expressed as total spike counts from all electrodes, were not significantly affected during the first 5–10 hours that followed CCh application (Figure 3A; see also Figure 4A), suggesting a qualitative change in network activity characteristics.

Several measures were used to quantify these qualitative changes. In the first we determined, for consecutive 10 ms bins, the number of active electrodes (that is, the number of electrodes from which an action potential was recorded at least once within that bin) and then calculated the distribution of active-electrodes values in 10 msec bins for each half-hour interval. The rational for this measure was that synchronous bursting would result in a broad distribution of active-electrode counts, with significant numbers of bins containing high counts (corresponding to burst peaks) and even larger numbers of bins containing near zero electrode counts (corresponding to interburst periods which tend to be almost entirely silent). Upon desynchronization, these extremes would be lost, resulting in a narrower distribution of active-electrode/bin values. CCh application was, as expected, associated with a loss of the highest active-electrode/bin counts (Figure 3C; logarithmic display) and a drastic reduction in the fraction of “silent” bins (Figure 3D; linear display).

A similar result was obtained using the “Burstiness Index” (BI) suggested by Wagenaar and colleagues ([39]; see Materials and Methods), which provides a single value measure of network synchronization within a given temporal window. As shown in Figure 3E, CCh application reduced the BI from a baseline value of ∼1.0 to <0.4.

Although the degree to which CCh suppressed synchronicity varied slightly among experiments, the effects were qualitatively similar in all experiments: For the first few hours following CCh application, overall activity levels were preserved (Figure 4A) whereas bursting was suppressed (Figure 4B). These short-term effects are in good agreement with prior reports [26]–[29]. It is worth noting, however, that the ability of CCh to suppress network synchronicity showed signs of wearing off with time (Figure 3B, two rightmost panels, Figure 4B, see also [28]). This matter will be readdressed later.

### Analysis of Carbachol-induced Synaptic Remodeling

The data presented so far show that experimental elevations of cholinergic tone lead to gradual synaptic growth, and that this growth does not result from a generalized suppression of network activity [30], [43], [44]. We have previously shown that by studying the remodeling history of many individual synapses, rules that govern synaptic remodeling can be uncovered. As shown in Figures 1C,D, the temporal and spatial resolution were sufficient to track individual PSD-95:EGFP puncta in consecutive images and measure changes in their fluorescence continuously over many hours and days (see also [30]). We therefore tracked individual puncta at 30 min intervals over 54 hour periods (27 hours before and after CCh application; see Video S2), including in our analysis only puncta that could be tracked reliably; puncta that split, merged or disappeared were rejected. While this probably biased the analysis toward the more stable synaptic population, it allowed us to explore potential rules underlying CCh-induced remodeling of persistent synapses.

**Figure 5:**
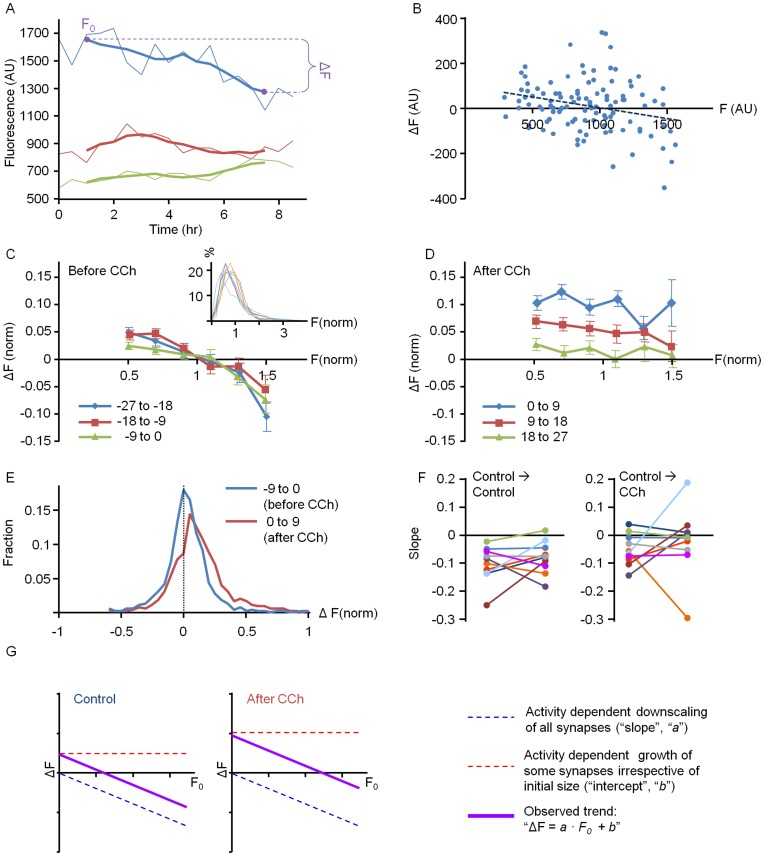
CCh-induced synaptic growth is not governed by multiplicative scaling or major changes in synaptic remodeling dynamics. ***A***) Example of fluorescence values for 3 PSD-95:EGFP puncta over a 9 hour time window (thin lines) and after smoothing with a 5-point (2 hour) low pass filter (thick lines). The calculation of *ΔF* and *F_0_* in subsequent figures is illustrated for the topmost trace. *ΔF* was calculated by subtracting the fluorescence at the beginning of each time window (F_0_) from the fluorescence at its end. As all such measurements were obtained from data smoothed with a 5 point filter, *F_0_* and *ΔF* were measured from the third data points from the beginning and the end of each 9 hour (18 data point) window. ***B***) *ΔF* vs. *F_0_* values for all tracked PSD-95:EGFP puncta in one site during a time window preceding CCh application. Note the significant changes in puncta fluorescence values (even after smoothing as showed in A) and a general trend which can be approximated by a linear regression fit with a negative slope that crosses that abscissa quite far from the origin. ***C,D***) Averaged *ΔF* over *F_0_* plots for pooled data (1087 puncta from 10 neurons from 5 separate experiments) before and after CCh application. Individual puncta of 2 neurons per experiment were followed for 54 hours (27 hours before and 27 hours after CCh application). To correct for cell to cell variability in PSD-95:EGFP expression levels, the fluorescence values of each neuron’s synapses were normalized to the average synaptic fluorescence of that neuron during the 27 hour period before CCh application (see Materials and Methods for further details). Thus, normalized *ΔF* and *F_0_* values are expressed as fractions of mean control fluorescence. As shown in *C* (inset), distributions of normalized synaptic fluorescence values in almost all neurons were very similar, indicating that this normalization procedure is appropriate. Data was then combined, binned (bin size  = 0.2 mean fluorescence units) and plotted, including only bins that contained at least 45 synapses (∼5% of the data). ***E***) Distribution of *ΔF* values for 9 hour windows before and after CCh applications. ***F***) Changes in the slopes of *ΔF* vs. *F_0_* regression fits between consecutive time windows before and after CCh application plotted for each cell separately (control → control: −27 to −18 vs. −18 to −9; control → CCh: −9 to 0 vs. 0 to 9). ***G***) Illustration of the two components presumed to give rise to the overall trends observed in *ΔF* vs. *F_0_* plots before (left) and after (right) CCh application. Data in *C,D*, mean ± SEM.

We first examined if and to what degree the change in PSD-95:EGFP fluorescence each synapse exhibited depended on its initial fluorescence. To that end, the 54 hour periods were broken down into 6 consecutive, 9 hour (18 time points) windows (3 before and 3 after CCh application). Then, *ΔF*, the change in fluorescence during each time window, was calculated by subtracting the fluorescence at its beginning (*F_0_*) from the fluorescence at its end, as illustrated in Figure 5A. *ΔF* was then plotted as a function of *F_0_*. As shown in Figure 5B, significant changes in puncta fluorescence over time were observed for most puncta, regardless of their initial size. In addition, and as we previously reported [30], stereotypical relationships between *F_0_* and *ΔF* were observed in most cells in the time windows preceding CCh application: Bright puncta tended to become dimmer whereas dim puncta tended to become brighter, as if activity was “constraining” the distributions of PSD sizes and driving their convergence to some optimal value. These relationships could be approximated reasonably well by linear regression fits that resulted in lines with negative slopes that intersected with the abscissae at some distance from the origin. It should be noted, that the *R^2^* values of these linear fits were not very high, indicating that these trends accounted for only a small portion of the remodeling exhibited by individual synapses. Nevertheless, these trends became unmistakable when data from all neurons and experiments analyzed in this fashion (1087 synapses, 10 neurons from 5 separate experiments) were normalized, pooled and binned (Figure 5C; see legend for details on data pooling).

After CCh application, relationships between *F_0_* and *ΔF* became less systematic (see below). Yet when all data was pooled, it became apparent that CCh led to a more or less uniform increase in mean PSD-95:EGFP puncta brightness regardless of initial puncta brightness (Figure 5D). In concordance with the population level analysis (Figure 2), this effect was most noticeable during the first 9 hour time window, and gradually diminished in subsequent time windows. This effect was not associated with concomitant increases in PSD-95:EGFP fluorescence in dendritic extra-synaptic regions (which actually showed a mean reduction of ∼10% over the same 27 hour period; data not shown), indicating that CCh-induced increases in puncta brightness were not the result of a generalized increase in neuronal PSD-95:EGFP levels. This analysis does not indicate that CCh–induced synaptic growth follows multiplicative scaling rules. In fact, it indicates that in relative terms, the smallest synapses showed the greatest fractional growth.

As we previously reported [30] stable distributions of synaptic sizes in unperturbed, active networks (such as those shown in Figure 2A,B) represent steady states of several processes. These include 1) the spontaneous, activity-*independent* remodeling of individual synapses (“dispersive” forces); and 2) activity-*dependent* processes that collectively tend to reduce the sizes of large synapses and increase the sizes of small synapses. As noted above, the latter “constraining” processes can be approximated reasonably well by linear regression fits in *ΔF* vs. *F_0_* plots (Figure 5B,C). Put differently, the relationships between *F_0_* and *ΔF* can be approximated by a linear function such that
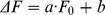
in which the magnitude of the slope *a* (typically a negative number) seems to mainly reflect the magnitude of a continuous, activity-dependent synaptic downscaling process, whereas the intercept *b* seems to represent an activity-dependent additive process that preferentially promotes growth in a subset of synapses irrespective of their initial size (illustrated schematically in Figure 5G; see also [30], [45]).

At steady state (stable synaptic size distributions) *a* and *b* are more or less constant, and *ΔF* vs. *F_0_* regression lines cross the abscissa at values that reflect the mean puncta fluorescence (unity, in normalized plots). Perturbations, however, that reduce the magnitude of *a* (attenuate activity-dependent synaptic downscaling processes) and/or increase the values of *b* (enhance activity-dependent synaptic growth processes) would lead to a broadening of synaptic size distributions in a manner similar to that seen in Figure 2. In addition, significant increases in ongoing synaptic remodeling dynamics would also have similar effects because these aforementioned “dispersive” processes are inherently asymmetric [30]. That is, synapses can grow larger but cannot grow smaller than zero (recall that synapses that disappeared were omitted from our analysis). Which of these, if any, did CCh perturb?

Starting with the latter, we estimated how CCh affected ongoing synaptic remodeling dynamics by calculating the range of changes in fluorescence explored by individual synapses [46]. In active networks, the fluorescence of ∼40% of all synapses changed by 20% or more over 9-hour time windows (Figure S1. See legend for details). These ranges were slightly but statistically significantly larger following CCh application (19.3%±11%, at −9 to 0 h versus 22.4±14% at 0 to 9 h, P<10^−6^, 1087 synapses from 10 neurons in 5 separate experiments, paired two tail t-test). Similarly, coefficient of variations (the standard deviation/mean of fluorescence values of each PSD95:EGFP punctum) were slightly but statistically significantly increased following CCh application (0.063±0.039 at −9 to 0 h versus 0.073±0.049 at 0 to 9 h, P<10^−6^, two tailed paired t-test). However, for reasons explained later, this seems more likely to be the result, rather than the cause, of the overall increases in synaptic PSD95:EGFP fluorescence values.

Where the slope *(a)* was concerned CCh did not seem to induce systematic changes. Although average slopes appeared shallower in *ΔF* vs. *F_0_* plots after CCh application, pairwise comparison of slopes for each site (as in Figure 5B) before and after CCh application failed to reveal systematic trends or statistically significant differences (P>0.49; two-tailed paired t-test; 10 neurons from 5 separate experiments); For some sites the slopes became shallower following CCh application while in others they did not, or even grew steeper (Figure 5F).

Our analysis, however did reveal a conspicuous increase in the intercept (*b*) in *ΔF* vs. *F_0_* plots (Figure 5D), which was statistically significant when compared on a site-to site basis (from 0.076±0.067 to 0.153±0.093 before and after CCh application, respectively, P<0.03; two tailed paired t-test). We interpret this to suggest that CCh caused net growth in a subset of synapses, the extent of which varied from one synapse to another (Figure 5E) but did not depend on the initial synapse size (Figure 5D).

We thus surmise that CCh-induced growth and broadening of PSD95:EGFP fluorescence distributions are not due to simple multiplicative scaling rules or large scale changes in spontaneous remodeling dynamics. Instead, as illustrated in Figure 5G, these seem to stem mainly from growth (potentiation? selective stabilization?) in a subset of synapses independent of their initial sizes.

### Carbachol Effects are Readily Reversible

As shown above, CCh reliably induced gradual growth of excitatory synapses while reducing the synchronicity of spontaneous network activity. To determine if an abrupt reduction in cholinergic tone would reverse these effects, we performed experiments similar to those described above, except that here, nine hours after CCh application, the neurons were exposed to a mixture of cholinergic antagonists (Atropine, 1 µM and Mecamylamine, 1 µM; see Materials and Methods for details), and recordings were continued for ∼50 to ∼70 hours.

**Figure 6:**
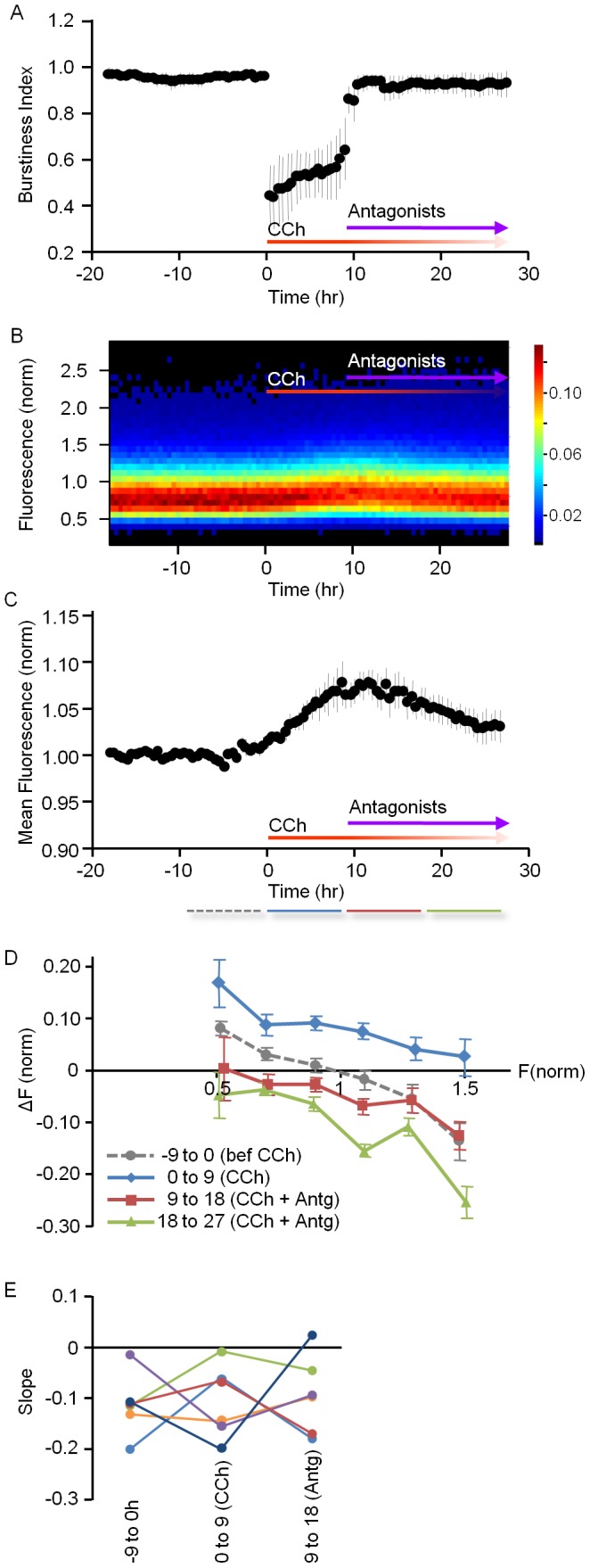
Rapid recovery of synchronous bursting and reversal of synaptic growth is observed after abrupt blocking of cholinergic receptors. 9 hours after the application of CCh, Atropine (1 µM), and Mecamylamine (1 µM) were added to the MEA dishes and the perfusion media. Data pooled from 3 experiments. ***A***) Burstiness index. ***B***) PSD-95:EGFP puncta fluorescence distribution (27 sites, 26 neurons) and ***C***) Mean PSD-95:EGFP puncta fluorescence. ***D***) Normalized, pooled and binned *ΔF* vs. *F_0_* plots for individually tracked synapses (674 puncta from 6 neurons from 3 separate experiments) before CCh (dashed line), after CCh application (blue line) and antagonist application (red and green lines). Note that both CCh and the antagonists mainly affected the intercepts of these plots, whereas the slopes were less affected. ***E***) Changes in the slopes of *ΔF* vs. *F_0_* linear regression fits between consecutive time windows before CCh, after CCh application and after antagonist application plotted for each cell separately. Note the lack of consistent trends. Data in *A,C,D*, mean ± SEM.

As before, CCh application drastically reduced synchronous bursting activity, induced a gradual broadening of PSD-95:EGFP puncta fluorescence distributions and increased mean PSD-95:EGFP puncta brightness (Figure 6A–C). Subsequent application of the aforementioned cholinergic antagonists was followed by a rapid reversal of these effects (three separate experiments, 27 sites, 26 neurons): Bursting activity was rapidly restored to pre CCh application levels (Figure 6A), PSD-95:EGFP puncta fluorescence distributions gradually constricted (Figure 6B), and mean PSD-95:EGFP puncta brightness gradually recovered (Figure 6C). In contrast, overall network activity levels were not significantly affected by CCh or subsequent antagonist application over these time scales (data not shown).

Tracking individual PSD-95:EGFP puncta allowed us to examine how the abrupt reduction in cholinergic tone affected the various measures of synaptic remodeling described above. We first examined the degree to which changes in PSD-95:EGFP fluorescence (*ΔF*) depended on their initial fluorescence values (*F_0_*.) as explained for Figure 5C,D. To that end we divided the data into 4 nine-hour windows, normalized, pooled, binned and plotted the data from all tracked synapses (674 synapses, 6 neurons from 3 separate experiments). As shown in Figure 6D, antagonist application led to a reduction in synaptic PSD-95:EGFP puncta fluorescence that was most pronounced for the brightest puncta, in particular at later time points. Put differently, a negative relationship between puncta fluorescence and the change it experienced in subsequent time points was apparent. While this relationship could be interpreted as a form of negative multiplicative scaling, closer examination, and in particular, comparison with the trends observed in times windows immediately preceding and following CCh application, indicates that the major antagonist-specific effect was a rather uniform reduction of PSD-95:EGFP fluorescence, irrespective of initial brightness. Antagonist application thus seemed to mainly affect (reduce) the value of the intercept (*b*), leaving the slope *(a)* more or less intact in *ΔF* vs. *F_0_* plots. Here again, comparison of such slopes before CCh application, after CCh application and after antagonist application at each of the 6 sites did not reveal systematic trends; in three cases shallower slopes observed after CCh application grew steeper again following exposure to antagonists; In the other three, however, the trends were either mixed or reversed (Figure 6E). Interestingly, the range of fluorescence values explored by individual synapses following exposure to antagonists did not decrease (if anything, it slightly increased; data not shown), which does not support the possibility that the antagonists suppressed ongoing synaptic remodeling; It thus seems likely that the changes in this measure observed here, as before, are the result, rather than the cause, of the overall decreases in synaptic PSD95:EGFP fluorescence values.

In summary, acute suppression of cholinergic tone by cholinergic antagonists led to a rapid recovery of synchronous bursting activity and a gradual reduction in synaptic sizes, in essence reversing the effects of CCh applications.

### Relationships between Measures of Synaptic Remodeling and Synchronous Bursting Activity

The manipulations of cholinergic tone described above led to rapid alterations in synchronous bursting activity, that were followed, on slower time scales, by changes in synaptic sizes. This raises the possibility that the changes in synaptic sizes were mediated by the changes in synchronous bursting activity. We observed some variability in the responses of different networks and neurons to CCh, both in the degree to which CCh suppressed bursting activity and in the various measures of synaptic remodeling described above. This variability allowed us to explore the quantitative relationships between these two major phenomena. To that end we examined relationships between the BI, and 1) changes in mean synaptic fluorescence for all cells in a particular experiment; 2) the slope (*a*) in *ΔF* vs. *F_0_* plots for individual sites; and 3) the intercepts (*b*) in these plots during the first 9 hours following CCh application.

**Figure 7:**
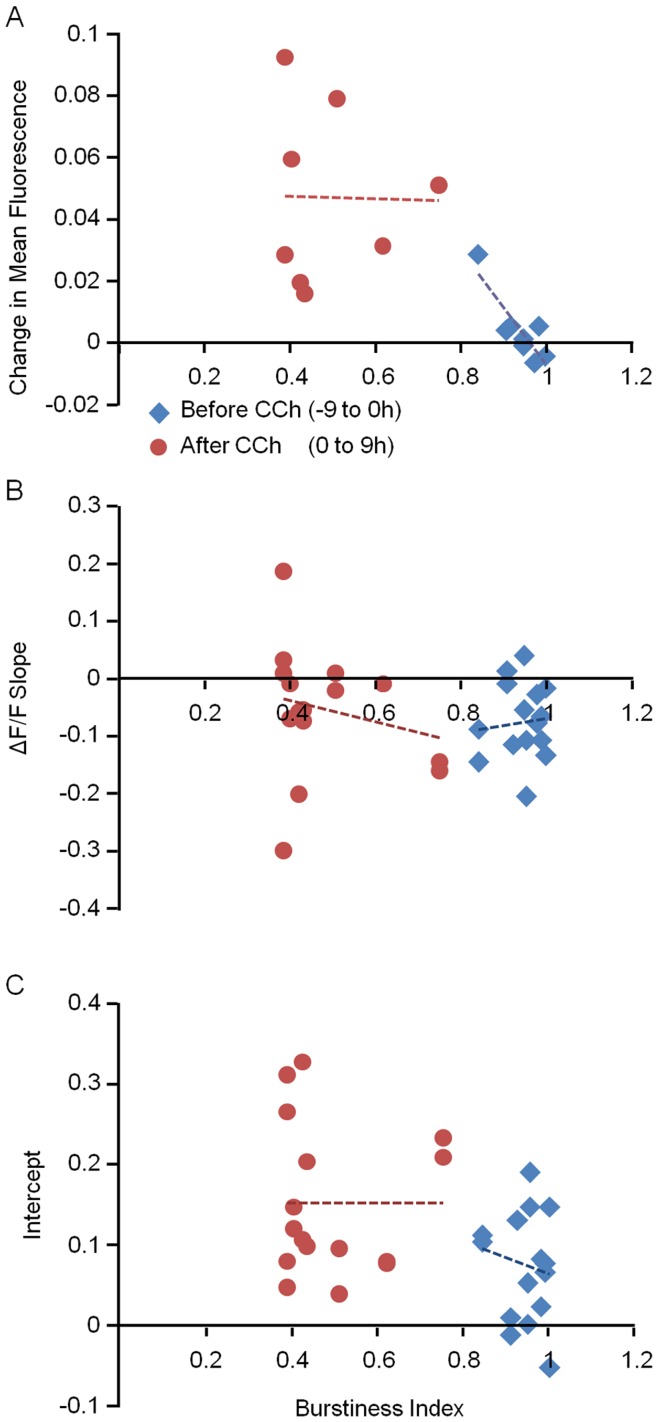
Synchronous bursting activity and measures of synaptic remodeling are not strongly correlated. Data from 8 different experiments (16 tracked sites) in 9 hour windows before (blue) and after (red) CCh application. Lines represent linear regression fits made separately for each group. The four panels show correlations between the burstiness index and ***A***) changes in mean synaptic PSD95:EGFP fluorescence (each point represents mean change in fluorescence of all synapses of all neurons in a certain experiment), ***B***) slopes of the *ΔF* vs. *F_0_* plots (each point represents data from tracked synapses from one site), and ***C***) the intercepts in the same plots.

Relationships between synchronous bursting and the aforementioned measures are shown in Figure 7. Data collected before and after CCh application was coded in different colors, and relationships were examined in each group separately. It should be noted that prior to CCh application, BI values tended to cluster near 1.0 and therefore relationships within this group were somewhat meaningless. This data was nevertheless provided to allow comparison with measurements made in the presence of CCh. As shown in this figure, no clear relationships were apparent in any of these comparisons within the CCh treated group during the first 9 hours following CCh application.

**Figure 8:**
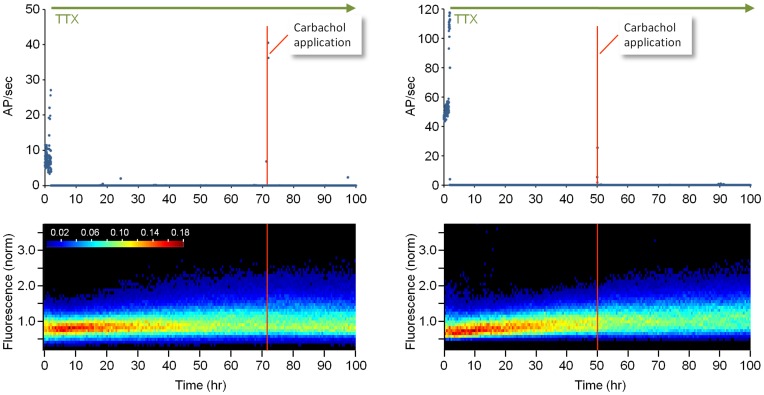
CCh does not induce synaptic growth in the presence of TTX. Two experiments in which TTX (1 µM) was added to the MEA dishes and the perfusion media 1–2 hours after the experiment was started. ***A***) Spontaneous activity in each network. ***B***) Synaptic PSD95:EGFP fluorescence distributions. Whereas TTX applications were followed by a significant broadening of these distributions, CCh had no further effect.

We also examined if CCh would lead to synaptic growth in preparations in which all activity was blocked. To that end we performed two experiments similar to those shown in Figure 4, except that in these experiments, TTX (1 µM) was added to the MEA dishes and the perfusion media 1–2 hours after the experiment was started. As expected, TTX application blocked all spontaneous activity and, as we previously reported [30], induced a significant broadening of synaptic PSD95:EGFP puncta fluorescence distributions. Interestingly, the addition of CCh after 50 or 72 h was not associated with further broadening of these distributions (Figure 8), suggesting that the effects of CCh on synaptic remodeling are activity dependent, although we cannot exclude the possibility that CCh-induced synaptic growth was occluded by the synaptic growth that followed TTX application.

**Figure 9:**
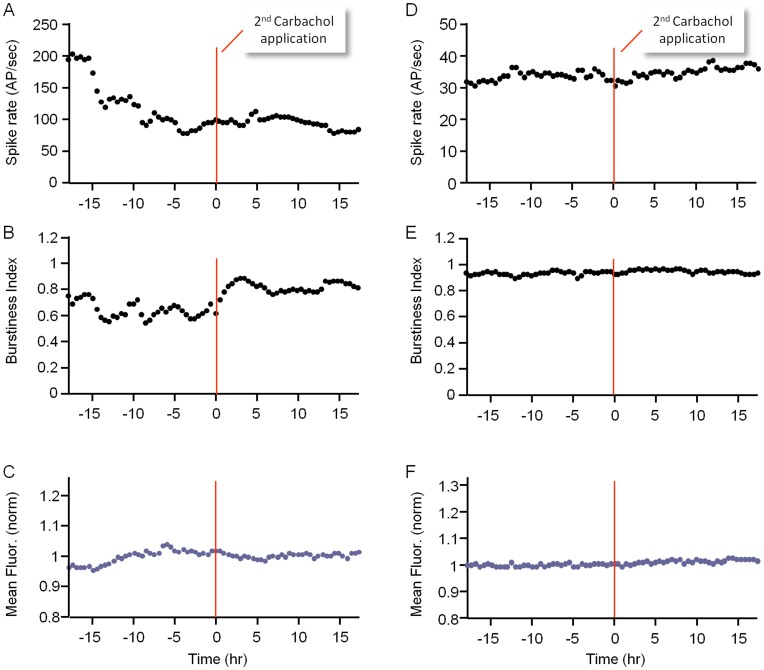
Second applications of freshly prepared CCh solutions do not affect activity characteristics or synaptic growth. Two experiments (out of three in total) in which a second bolus of freshly prepared CCh solution was added to the MEA dishes 143 h (left, same experiment as Figure 1) and 137 hours (right) after the experiment was started (45 and 89 hours after first CCh application, respectively). ***A***) Spontaneous activity rates. ***B***) Burstiness index. ***C***) Mean synaptic size (left - 5 sites; right - 8 sites).

As mentioned briefly above, we noted that the initial suppression of synchronous bursting activity by CCh was followed by a slow recovery of such bursting, which gradually returned to dominate network activity (Figures 3, 4, 6). This recovery began, at most, 12 hours after CCh application, and occurred even though CCh was continually present in the perfusion media. We were initially concerned that this might be due to the gradual breakdown of CCh in the MEA dish or in the perfusion media. We reasoned that if this is the case, a second bolus of freshly prepared CCh solution should resuppress synchronous bursting activity and increase mean synaptic PSD-95:EGFP fluorescence. In 3 experiments, however, in which freshly prepared CCh solution was added to the MEA dish at various times after the first application (25, 45, 89 h), no such effects were observed (Figure 9). These findings thus strongly argue against the possibility that the gradual reappearance of synchronous bursting is due to the gradual breakdown of CCh.

**Figure 10:**
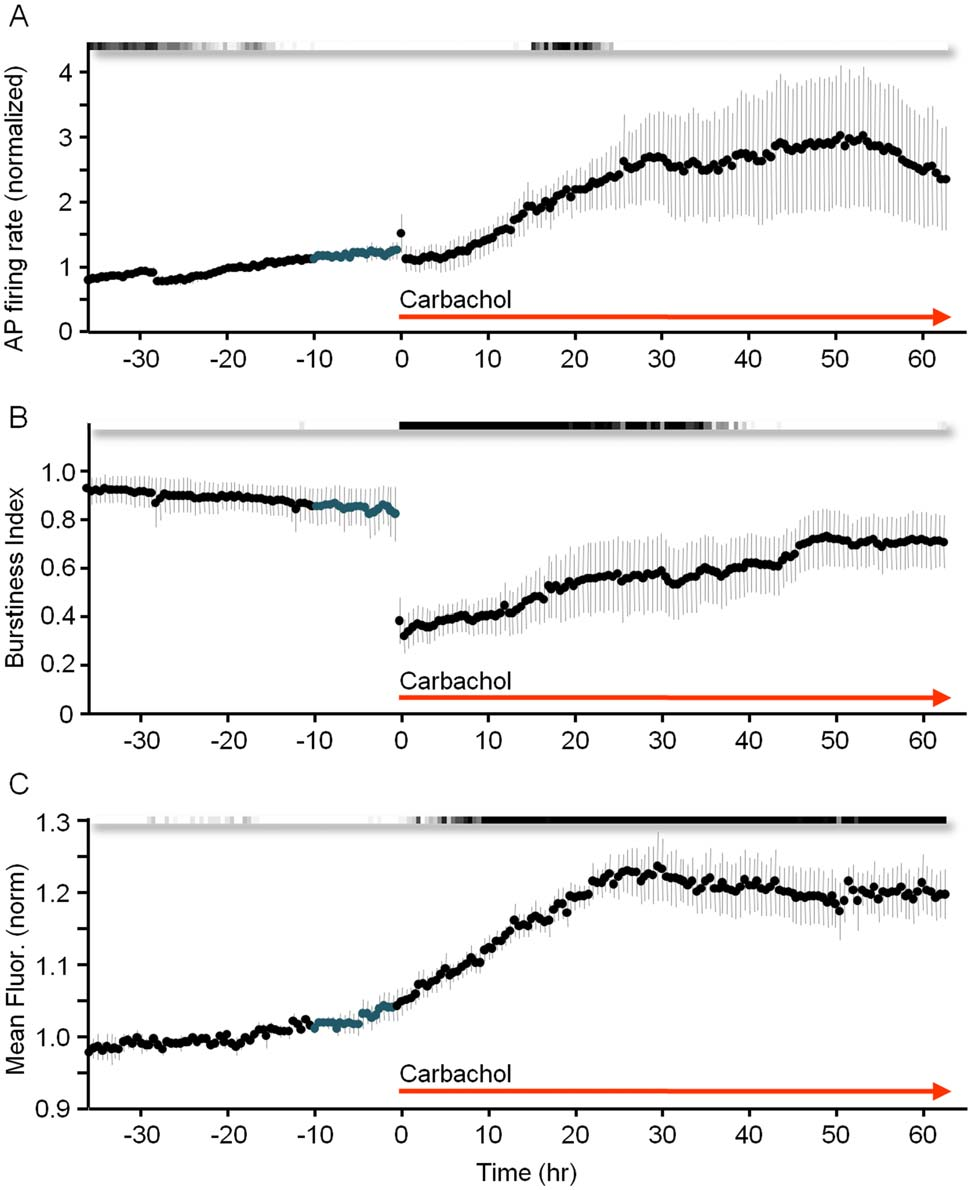
Gradual recovery of synchronous bursting activity but not mean synaptic size in the continual presence of CCh. Pooled data from 4 experiments from 27 hours before to 63 hours after CCh application. ***A***) Firing rates recorded from all 59 electrodes, normalized to average firing rates during the 27 hour time window before CCh application. ***B***) Burstiness index. ***C***) Mean PSD-95:EGFP puncta fluorescence normalized separately in each experiment to average puncta fluorescence during the 27 hour time window preceding CCh application. Grayscale bars indicate statistical significance as in Figure 2F. Data shown as mean ± SEM.

To further explore the relationships between synaptic remodeling and synchronous bursting, we carried out very long recordings (>60 hours) in the continual presence of CCh, to examine whether the recovery of synchronous bursting will ultimately affect (reduce) synaptic sizes. As shown in Figure 10 (4 separate experiments) the recovery of synchronous bursting activity over these time scales was practically complete, with bursting no longer significantly different from pre-CCh levels after ∼40 hours (Figure 10B). PSD95:EGFP puncta fluorescence values in these experiments, however, remained elevated, and failed to recover even after many hours of intense synchronous bursting activity (Figure 10C).

Collectively these findings do not support a tight association between CCh-induced synaptic remodeling and measures of network activity synchronicity. As CCh-induced synaptic remodeling did seem to depend on activity, these findings indicate that CCh-induced synaptic growth depends on finer features of neuronal activity not captured by the global measures of network activity used here.

## Discussion

Here we describe experiments aimed at evaluating long-term relationships between cholinergic tone, synaptic remodeling and network activity characteristics. We found that CCh application was followed by an abrupt suppression of synchronous bursting activity and a gradual growth of excitatory synapses. Both effects were reversed by subsequent blockage of cholinergic receptors. Neither synaptic growth nor downsizing seemed to follow multiplicative scaling rules. Instead, CCh seemed to promote net growth in a subset of synapses, irrespective of their initial size. CCh-induced synaptic growth seemed to depend on intrinsic network activity, but not on the degree to which bursting was suppressed. Intriguingly, sustained elevations of cholinergic tone were associated with a gradual recovery of synchronous bursting but not with a reversal of synaptic growth, even many hours after synchronous bursting had recovered to near baseline levels. These findings thus show that cholinergic tone can strongly affect both synaptic remodeling and synchronous bursting, but do not support a strict coupling between the two.

### Studying Relationships between Cholinergic Tone, Synaptic Remodeling and Synchronous Bursting in ex-vivo Networks

Whereas the combination of techniques described here allowed us to study relationships between cholinergic tone, synaptic remodeling and network activity characteristics in exquisite detail, this approach has certain shortcomings that warrant discussion.

One concern relates to the temporal structure of cholinergic tone manipulation. Unlike the tonic CCh levels that neurons experienced in our experiments, neurons *in vivo* experience cholinergic levels that vary with phasic discharge patterns of cholinergic neurons, acetylcholine degradation and clearance rates as well as spatial relationships with cholinergic release sites. Indeed, recent studies suggest that synaptic plasticity is sensitive to the precise timing of cholinergic neuron activity ([5] and references therein). The implications of such precise timings for regimes of continuous and complex activity patterns are not clear, in particular when taking into account the tendency of basal forebrain cholinergic neurons to discharge in ∼7 Hz rhythmic bursts [47] and the relatively slow kinetics of neuronal muscarinic acetylcholine receptors [48]. Nevertheless, we cannot exclude the possibility that some of our findings were affected by the tonic nature of cholinergic tone manipulation.

A second, more general concern relates to the model system used here, that is, networks of dissociated rat cortical neurons maintained in primary culture. Beyond obvious differences such as anatomy, cellular makeup, developmental status and bodily context, these networks receive no input from the external world or from other brain regions. On the one hand, it is this very detachment that allowed us to explore relationships between cholinergic tone, synaptic remodeling and network activity characteristics in a generic manner, free from the influences of other intrinsic neuromodulators, circadian systems and external stimuli. On the other, the absence of input from the external world means that network activity characteristics probably fail to accurately mimic activity patterns in the intact brain. It should be noted, however, that intrinsically generated activity has been hypothesized to play crucial roles in brain circuitry maturation during REM sleep [10], a predominant brain state during development and early life, that is both associated with [49], [50] and controlled by elevated cholinergic tone [8], [9]. Indeed, our observations that CCh-induced synaptic growth occurred in our reduced system without any input from the external world might be taken to indicate that synaptic remodeling, driven by elevated cholinergic tone and intrinsic activity, is an inherent property of cortical neuronal networks whether or not sensory input is being processed.

### Relationships between Cholinergic Tone, Synaptic Remodeling and Synchronous Bursting

Reduced cholinergic (and monoaminergic) tone during the predominant form of adult mammalian sleep, known as slow wave sleep, is thought to trigger a striking form of neocortical activity known as ‘slow rhythmic’ or ‘slow wave’ activity. This form of activity is characterized by bursts of neuronal discharges separated by periods of nearly complete quiescence (associated with so called ‘up’ and ‘down’ states), that occur nearly synchronously in large cortical domains [7], [51], [52]–[60]. As mentioned in the introduction, sleep is also associated with reductions in synaptic numbers, sizes and strengths. It has been hypothesized that these sleep-associated synaptic downsizing processes are driven by ‘slow wave’ activity, which counterbalances synaptic growth associated with sensory experiences during waking and thus prevents spiraling, unsustainable synaptic growth [61]. Although synchronous bursting patterns observed in *ex-vivo* cortical networks do not fully represent slow wave sleep associated activity forms, these *in vivo* and *ex vivo* forms of spontaneous, episodic bursting phenomena share many similarities (reviewed in [62]). Most notably, in the present context, both *in-vivo* [6]–[9] and *ex vivo* forms (Figures 3, 4, 6,7, 10; see also [26]–[29]) are strongly suppressed by elevated cholinergic tone. Assuming that synchronous bursting in our preparations shares certain features with ‘slow wave’ activity, we might have expected to observe some of the predicted relationships between synchronous bursting and synaptic remodeling [61]. However, such relations were not found, at least during the initial period following CCh application (Figure 7), nor did we find evidence that prolonged bursting in the presence of CCh induced synaptic downsizing (Figure 10). Our findings are thus more consistent with the possibility that the synaptic growth and downsizing we observed in electrically active networks were mainly governed by cholinergic tone rather than synchronous bursting *per-se*.

A recent study [63] demonstrated that applications of a muscarinic agonist can drive a rapid and reversible emergence of fine filopodia from spine heads of hippocampal neurons. Interestingly, the emergence of spine head filopodia was not suppressed by TTX, or blockers of glutamatergic transmission; furthermore, spine head filopodia formation did not occur in response to experimentally-induced epileptiform activity. Although we did not observe this phenomenon in the current study, this might have been precluded by the use of a PSD reporter molecule as compared to the membrane targeted reporter used in that study. Nevertheless, this report further supports the capacity of elevated cholinergic tone to directly affect synaptic remodeling.

### Cholinergic Tone and Spontaneous Synchronous Bursting–a Hypothesis

In practically all experiments, we noted that the initial CCh-induced suppression of synchronous bursting was followed by a gradual recovery of bursting activity. This recovery became apparent after 12 hours at most (Figures 3, 4, 6), with synchronous bursting returning to near baseline levels after ∼40 hours (Figure 10B). As this was not due to CCh breakdown (Figure 9), this recovery might have been driven by various adaptation and homeostatic processes (see below). Alternatively, the gradual increase in synaptic sizes (Figures 2, 6, 10), which presumably increases synaptic strengths, might have increased the propensity for synchronous bursting [61]. Regardless of the underlying mechanisms, these observations indicate that the capacity of chronically elevated cholinergic tone to suppress synchronous bursting indefinitely might be inherently limited.

This finding could have potentially important implications for understanding general relationships between cholinergic tone and synchronous bursting: Although episodic synchronous bursting is a well known characteristic of neuronal networks of dissociated cortical neurons, it is by no means unique to this preparation. Episodic, synchronous bursting occurs in acute and organotypic cortical preparations (e.g. [64]–[67]), during prenatal and postnatal development (e.g., [68]–[73]), in animals under anesthesia (e.g. [52], [53], [74]), and, as mentioned above, during slow wave sleep. It is now well established that the suppression of slow wave activity during the transition to the wake (and REM sleep) state is largely driven by elevations in cholinergic tone [8], [9]. However, if the capacity of cholinergic neuromodulation to suppress synchronous bursting is inherently limited, some degree of synchronous bursting might be expected to eventually reappear. Indeed, in behaving rodents, epochs of slow wave activity start showing up near the end of wake periods, especially if sleep has been prevented ([51], [59] and references therein).

The reappearance of synchronous bursting not only in culture but *in vivo* as well raises possibilities, which although speculative, are quite intriguing. One relates to the very nature of synchronized bursting activity. For example, the prevalence of synchronized bursting activity both *in vivo* and *in vitro*, as described above, might be taken to indicate that this is, in some sense, a ‘default’ mode which is inherent to cortical [52], [53], [55], [56], [74] and probably other [62], [75] neuronal networks when these are deprived of input [67], [76] as well as neuromodulatory influences (e.g. [77]). From this perspective, the neuromodulatory systems that act during waking [8] might be viewed as systems that among their myriad activities, prevent cortical networks from collapsing into this ‘default’ mode by tuning and maintaining neuronal membrane excitability and synaptic properties within functionally optimal limits [77]. If this capacity is inherently limited, however, due to receptor desensitization [78], internalization and degradation and, perhaps, slower homeostatic processes [42], [43], [44], [71], [79], [80], the ability of neuromodulatory systems to counter the gravitation toward ‘default’ bursting modes would gradually diminish in a manner comparable, perhaps, to what we have observed here (Figures 3, 4, 6, 10). We hypothesize that the time-limited efficacy of (cholinergic) neuromodulation might have been ‘addressed’ in several manners. The first is a multiplicity of neuromodulatory systems, providing more degrees of freedom for tuning network characteristics [8], [74]. The second is a periodic (and tightly controlled) withdrawal of neuromodulators, essential for allowing the system to recover its capacity to respond to these substances during the next cycle. If this hypothesis is correct, then these withdrawal periods, manifested as periods of slow-wave sleep, might be regarded as the unavoidable price of staying awake.

## Materials and Methods

### Cell Culture

Primary cultures of rat cortical neurons were prepared as described previously [30] using a protocol approved by the Technion committee for the supervision of animal experiments. Briefly, cortices of 1–2 days-old Sprague-Dawley rats were dissected, dissociated by trypsin treatment followed by trituration using a siliconized Pasteur pipette. 1–1.5*10^6^ cells were then plated on thin glass Multielectrode array (MEA) dishes (MultiChannelSystems MCS, Reutlingen, Germany) whose surface had been pre-treated with Polyethylenimine (Sigma, St. Louis, MO) to facilitate cell adherence. Cells were initially grown in media containing minimal essential medium (MEM, Sigma), 25 mg/l Insulin (Sigma), 20 mM Glucose (Sigma), 2 mM L-Glutamine (Sigma), 5 µg/mL Gentamycin sulfate (Sigma) and 10% NuSerum (Becton Dickinson Labware, Bedford, Massachusetts, United States). The preparation was then transferred to a humidified tissue culture incubator and maintained at 37°C in a gas mixture of 5% CO_2_, 95% air. Half the volume of the culture medium was replaced 3 times a week with feeding media similar to the media described above but devoid of NuSerum, containing a lower L-Glutamine concentration (0.5 mM) and 2% B-27 supplement (Invitrogen, San Diego, CA).

### Lentivirus Production and Transduction of Cortical Cultures

Lentiviral particles were produced using a mixture of FU(PSD-95:EGFP)W [30] and the Lentiviral packaging vector mix of the ViraPower four plasmid lentiviral expression system (Invitrogen). HEK293T cells were co-transfected with a mixture of FU(PSD-95:EGFP)W and the three packaging plasmids - pLP1, pLP2, pLP\VSVG. Transfection was performed in 10 cm plates when the cells had reached 80% confluence, using 3 µg of FU(PSD-95:EGFP)W, 9 µg of the packaging mixture and 36 µL of Lipofectamine 2000 (Invitrogen). Supernatant was collected after 48 h, filtered through 0.45 µm filters, aliquoted and stored at −80°C. Transduction of cortical cultures was performed on day 5 *in-vitro* by adding 10 µL of the filtered supernatant to each MEA dish.

### Long-term Imaging

All imaging was performed on neurons grown on thin glass MEA dishes as described above. These particular MEA dishes are fabricated of very thin glass (180 µm), which allows for the use of high numerical aperture, oil immersion objectives and are thus ideally suited for high-resolution imaging [30]. Scanning fluorescence and brightfield images were acquired using a custom designed confocal laser scanning microscope based on a Zeiss Aviovert 100 using a 40×, 1.3 N.A. Fluar objective. The system was controlled by software written by one of us (NEZ) and includes provisions for automated, multisite time-lapse microscopy. MEA dishes containing networks of neocortical neurons were mounted on a commercial 60-channel headstage/amplifier (see below) attached to the microscope’s motorized stage. The MEA dish was covered with a custom designed cap containing inlet and outlet ports for perfusion media and air mixtures, a reference ground electrode and a removable transparent glass window. The MEA dish was continuously perfused with feeding media (described above) at a rate of 2.5 ml/day by means of a custom built perfusion system based on an ultra slow flow peristaltic pump (Instech Laboratories Inc., Plymouth Meeting, PA, USA) using an imbalanced set of silicone tubes. The tubes were connected to the dish through the appropriate ports in the custom designed cap. A 95% air/5% CO_2_ mixture was continuously streamed into the dish at very low rates through a third port with flow rates regulated by a high precision flow meter (Gilmont Instruments, IL, USA). The base of the headstage/amplifier and the objective were heated to 37°C and 36°C respectively using resistive elements, separate temperature sensors and controllers, resulting in temperatures of 36–37°C in the culture media.

EGFP was excited using the 488 nm line of an argon laser. Fluorescence emissions were read through a 500–545 nm bandpass filter (Chroma Technology, Brattleboro, VT). Time-lapse recordings were usually performed by averaging six frames collected at each of 9 to 11 focal planes spaced 0.8 µm apart. All data were collected at a resolution of 640×480 pixels, at 12 bits/pixel, with the confocal aperture fully open. Data was collected sequentially from up to 12 predefined sites, using the confocal microscope robotic XYZ stage to cycle automatically through these sites at 30 minutes time intervals. Focal drift during the experiment was corrected automatically by using the microscopes’ “autofocus” feature.

### Electrophysiology

The thin glass MEA dishes used here contained 59, 30 µm diameter, electrodes arranged in an 8×8 array, spaced 200 µm apart. The dishes contain 59 rather than 64 electrodes because the corner electrodes are missing, and one of the remaining leads is connected to a large substrate embedded electrode designed to be used as a reference (ground) electrode. The flat, round (30 µm diameter) electrodes are made of titanium nitride, whereas the tracks and contact pads are made of transparent Indium Tin Oxide. The aforementioned reference electrode was not used here, and instead, a submerged platinum wire loop connected to the custom designed cap (see above) was used.

Network activity was recorded through a commercial 60-channel headstage/amplifier (Inverted MEA1060, MCS) with a gain of 1024× and frequency limits of 1–5000 Hz. The amplified signal was multiplexed into 16 channels, amplified by a factor of 10 by a 16 channel amplifier (Alligator technologies, Costa Mesa, CA, USA) and then digitized by an A/D board (Microstar Laboratories, WA, U.S.A.) at 12 KSamples/sec per channel. Data acquisition was performed using AlphaMap (Alpha-Omega, Nazareth, Israel). All data was stored as threshold crossing events with the threshold set to −40 µV. Electrophysiological data were imported to Matlab (MathWorks, MA, USA) and analyzed using custom written scripts.

### Pharmacological Manipulations

CCh (Carbachol, Carbamoylcholine), cholinergic receptor antagonists Mecamylamine and Atropine (Sigma-Aldrich, Israel) and Tetrodotoxin (TTX, Alomone labs, Israel) were applied by diluting them into 100 µL of medium drawn from the culture dish while on the microscope. The mixture was subsequently returned to the dish and mixed gently. Applications to the dish were complemented by simultaneous addition to the perfusion media. Final concentrations in the dish and perfusion media were 20–50 µM (CCh), 1 µM (Mecamylamine), 1 µM (Atropine) and 1 µM (TTX).

In experiments in which Mecamylamine and Atropine were used, these were added to the culture dish and to CCh-free perfusion media. As we have previously noted that rapid washes and complete media replacements are rather strong perturbations in their own right (often leading to transient changes in network activity and synaptic characteristics, data not shown), we used cholinergic antagonists rather than thorough washes with CCh-free media in order to reduce cholinergic tone.

### Data Analysis-imaging

All imaging data analysis was performed using custom written software (“OpenView”) written by one of us (NEZ). Special features of this software allow for automated/manual tracking of objects in 3D time series of confocal images. Fluorescent puncta were located by 1) smoothing the data with a 3×3 low pass filter; 2) identifying pixels whose fluorescence exceeded that of neighboring pixels by a predefined value; 3) merging peak pixels with common borders into one object, and 4) calculating the center of mass of such objects. This procedure correctly identifies almost all discernable puncta, from the very dimmest to the very brightest, even for nearby puncta whose fluorescence partially overlaps. 9×9 pixel (∼1.3×1.3 µm) areas were then centered on the centers of such objects and mean pixel intensities within these areas were obtained from maximal intensity projections of Z section stacks. For measuring distributions of puncta intensities (such as those of Figure 2), areas were placed programmatically at each time step using identical parameters but no tracking of individual puncta was performed. For tracking identified puncta, areas were placed initially over all puncta and then a smaller subset (typically 100–150 per site) was thereafter tracked (Video S2). Automatic tracking was based on weighted comparisons of vicinity (in X,Y and Z), on intensity, and most importantly, on “constellations” that is, punctum location relative to neighbouring puncta within a radius of 50 pixels. As the reliability of automatic tracking was not absolutely perfect, all tracking was verified and, whenever necessary, corrected manually. Puncta for which tracking was ambiguous (as explained in main text) were excluded. To minimize the effects of short term fluctuations and measurement noise, fluorescence measurements made for each punctum were first smoothed using a 5 point (2 hour) low pass filter and this smoothed data was used for all subsequent analysis.

Range of fluorescence changes explored by individual synapses was quantified as described previously [46]. Briefly for each synapse, the “range/mean” values was calculated according to the following equation
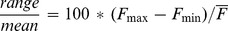
where *F_max_* is the maximal fluorescence measured for a given synapse during a time window, *F_min_* the minimal fluorescence and 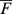 the mean fluorescence values for the same period. All values were obtained from the low-pass filtered data as illustrated in Figure S1A.

All data were exported to Matlab and analyzed using custom written scripts. Microscopy images for Figure 1 were processed by contrast enhancement and low-pass filtering using Adobe Photoshop. Final graphs were prepared using Microsoft Excel. All final figures were prepared using Microsoft PowerPoint.

### Data Analysis-electrophysiology

Distributions of active electrodes/bin were obtained by counting the number of active electrodes (that is, the number of electrodes from which an action potential was recorded at least once within that bin) in consecutive 10 ms bins, and then calculating the distribution of active-electrodes/bin values for each half hour interval.

Burstiness index was calculated according to Wagenaar and colleagues [39] with some adjustments of parameters. Each half-hour interval of data was divided into consecutive 100 ms bins, and action potentials were counted per bin. The fraction (*f_15_*) of the number of action potentials accounted for in the top 15% of bins (those with largest count values) out of the total number of action potentials in that interval was calculated and normalized between 0 to 1 to give the burstiness index (BI):
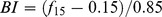



This index is largely independent of firing rate, as it does not impose an absolute threshold in terms of action potential count per bin in order to detect bursting activity. However, it does depend on the fraction (here defined as 15%) of the highest counts representing burst dominated bins, and therefore, to some extent, on the burst rate. As our networks were usually bursting at a frequency of 0.1–1 Hz with each burst lasting approximately 100–500 ms, most of the burst-dominated bins should be within the 15% fraction.

## Supporting Information

Figure S1 **Range of changes explored by individual synapses.** ***A*** **)** Illustration of the measure used for calculating the range of changes explored by individual synapses (“range/mean”) [46]. Trace shown is from Figure 1A (middle trace). ***B*** **)** Distribution of range/mean values for 18 consecutive hours before and after CCh application. As the distributions were clearly not normally distributed, the paired two-tail t test mentioned in the main text was performed after a logarithmic transformation of range/mean values, as this transformation resulted in approximately normal distributions of the transformed values. A small but statistically significant increase in range/mean values was observed after CCh application (19.3%±11%, at −9 to 0 h vs. 22.4±14% at 0 to 9 h, P<10^−6^, 1087 synapses from 10 neurons in 5 separate experiments)
(TIF)

Video S1 **3-day long time-lapse of dendritic spines on several dendrites belonging to one neuron expressing PSD-95:EGFP.** This video contains 150 frames (maximum intensity projection of 10 sections, 0.8 µm apart) collected at 30 min intervals (75 hours in total) starting 71 hours (∼3 days) after the beginning of the experiment. CCh (20 µM) was applied 27 hours (cycle 196 in video) into the video. Apart from slight contrast enhancement, the images are unprocessed. Image quality is substantially degraded, however, due to the compression involved in preparing the video. The black margins surrounding the image are due to the inter-frame alignment required to compensate for minor positioning inaccuracies of the motorized XY stage. Cycle number, running time and presence of CCh are indicated in left top corner. Same experiment as that shown in Figures 2A–E, 3.
(WMV)

Video S2 **Tracking individual PSD-95:EGFP puncta.** Same time-lapse video as in Video S1, except that here, 101 puncta that were tracked throughout this period are surrounded by blue open squares. Note that these squares (13×13 pixels) are larger than those used to measure the fluorescence of the tracked puncta (see Materials and Methods). Puncta whose intensities approached saturation were ignored.
(WMV)
