# Supplementary figures and images for: Long-term Relationships between Cholinergic Tone, Synchronous Bursting and Synaptic Remodeling
Source: PLoS One. 2012 Jul 23;7(7):e40980. doi: 10.1371/journal.pone.0040980 (PMC3402441; doi:10.1371/journal.pone.0040980)

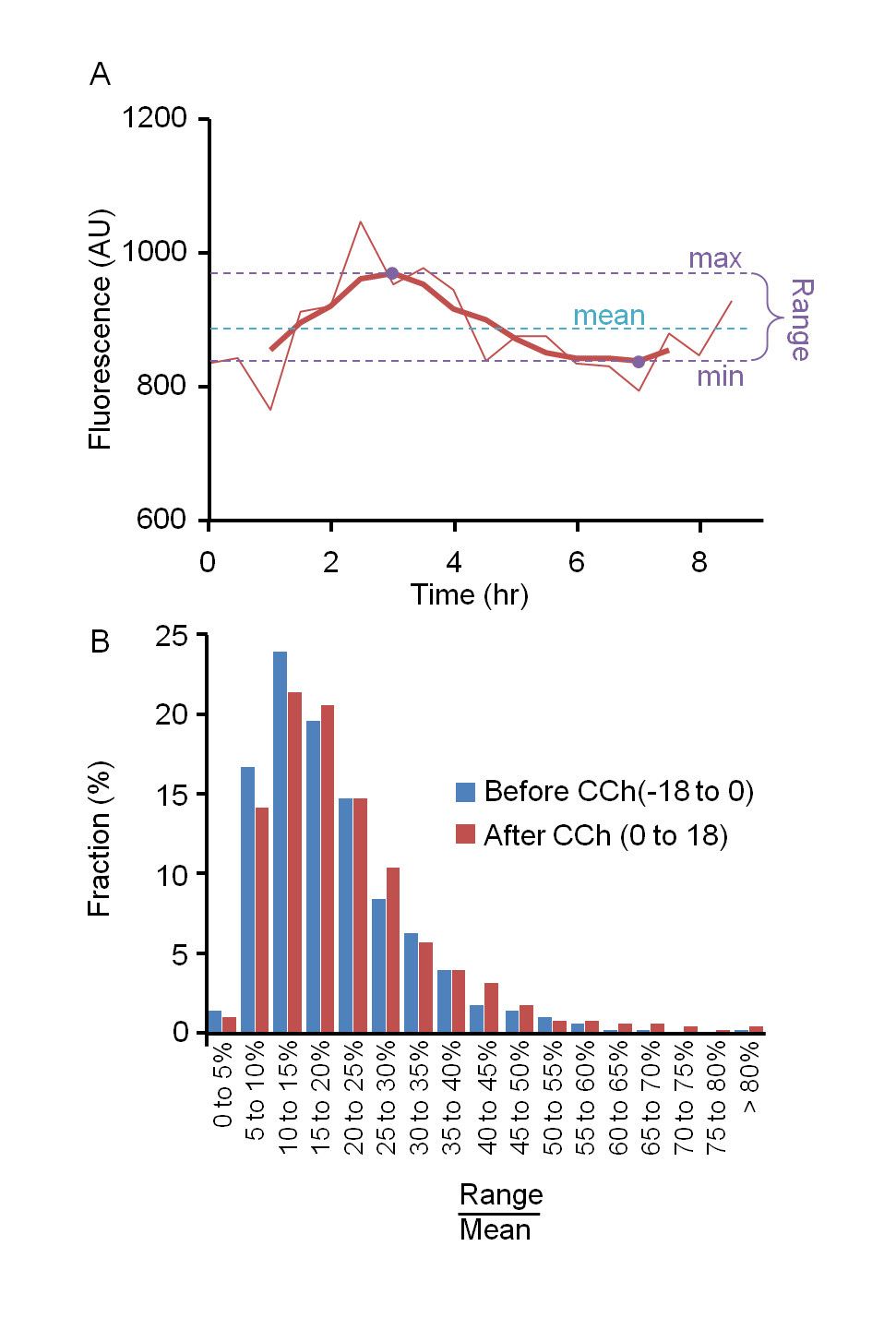

Supplement: Figure S1 — Range of changes explored by individual synapses. A ) Illustration of the measure used for calculating the range of changes explored by individual synapses (“range/mean”) [46]. Trace shown is from Figure 1A (middle trace). B ) Distribution of range/mean values for 18 consecutive hours before and after CCh application. As the distributions were clearly not normally distributed, the paired two-tail t test mentioned in the main text was performed after a logarithmic transformation of range/mean values, as this transformation resulted in approximately normal distributions of the transformed values. A small but statistically significant increase in range/mean values was observed after CCh application (19.3%±11%, at −9 to 0 h vs. 22.4±14% at 0 to 9 h, P<10−6, 1087 synapses from 10 neurons in 5 separate experiments) (TIF) [file pone.0040980.s001.tif]
